# Molecular Epidemiology of Human Norovirus in Korea in 2013

**DOI:** 10.1155/2015/468304

**Published:** 2015-09-02

**Authors:** Jae-Seok Kim, Hyun Soo Kim, Jungwon Hyun, Han-Sung Kim, Wonkeun Song

**Affiliations:** Department of Laboratory Medicine, Hallym University College of Medicine, 7 Keunjaebong-gil, Hwaseong-si, Gyeonggi-do 445-170, Republic of Korea

## Abstract

Norovirus is a major cause of acute gastroenteritis. The molecular epidemiology of norovirus exhibits temporal and geographical fluctuations, and new variants of the GII.4 genotype emerge every 2-3 years to cause global epidemics of acute gastroenteritis. We investigated GI and GII genotypes of human norovirus strains isolated from patients with acute gastroenteritis in Korea in 2013. Norovirus antigen test was performed on 2,980 fecal specimens from January to December 2013. RNA was extracted from norovirus antigen-positive fecal suspensions, and the norovirus capsid (VP1) and polymerase (RdRp) genes were characterized by RT-PCR and sequencing. Of the 230 genotyped strains, GII.4 (77.3%) was the most frequently observed capsid genotype, followed by GII.3 (6.1%) and GII.13 (3.9%). A norovirus GII.4 variant, GII.Pe/GII.4 Sydney 2012, was the most frequently found polymerase/capsid genotype (65.7%), followed by GII.P17/GII.17 (2.1%) and GII.P21/GII.3 (2.1%). Phylogenetic, similarity, and capsid epitope analyses of GII.Pe/GII.4 Sydney 2012 strains were performed. We concluded that the norovirus GII.4 variant, GII.Pe/GII.4 Sydney 2012, was the main cause of norovirus-related gastroenteritis in Korea in 2013.

## 1. Introduction

Noroviruses are the primary cause of epidemic and sporadic acute infectious gastroenteritis among people of all ages worldwide [1–4]. Norovirus belongs to the family Caliciviridae and has a positive-sense, single-stranded RNA genome of 7.5~7.7 kb. The genome consists of three open reading frames (ORFs). ORF1 encodes nonstructural proteins such as NTPase, protease, and RNA-dependent RNA polymerase (RdRp). ORF2 encodes the major capsid protein (VP1) and ORF3 encodes the minor capsid protein (VP2). There are six major norovirus genogroups (GI–GVI), defined according to major capsid protein VP1 sequences [2, 5]. GI and GII are the most frequently detected genogroups in human infections and GIV is also implicated in human gastroenteritis. Genogroups can be subdivided into genotypes, of which 9 GI and 23 GII capsid genotypes have been described [1, 5].

However, genotyping based merely on capsid sequence variations is not sufficient to characterize norovirus because the norovirus genome frequently undergoes recombination, leading to different capsid and polymerase gene types. Thus far, 9 GI and 23 GII capsid genotypes and 14 GI and 29 GII polymerase genotypes have been described and the current nomenclature comprises both polymerase and capsid genotypes [1, 5].

The genotypic distribution of norovirus strains showed temporal and geographical fluctuations [1–3]; thus continuous monitoring is necessary to prevent and control the spread of norovirus outbreaks. Molecular epidemiology data suggest that GII.4 genotype and its variants are the most frequent genotypes, evolve rapidly, and have been associated with many norovirus outbreaks. New GII.4 variants emerge every 2-3 years and become the cause of global epidemics of acute gastroenteritis, including US 1995/96 in the late 1990s, Farmington Hills virus in 2002, Hunter virus in 2004, Den Haag 2006b virus in 2007-2008, New Orleans virus 2009 in 2009–2012, and most recently Sydney 2012 in 2012-2013 [6]. Sequence and structural characterization of the antigenic properties of capsid protein have revealed that the surface-exposed subdomain P2 (residues 279 to 405) interacts with potential neutralizing antibodies and norovirus carbohydrate-binding ligands and that changes in highly variable sites (epitopes A to E) within the P2 subdomain of GII.4 norovirus correlate with the emergence of new epidemic strains [7].

In South Korea, norovirus infection is most prevalent from November to January [8]. One study found that GII.1 was most frequent in Gyeonggi from 2001 to 2005 [9], whereas GII.4 was most frequently identified in most other studies [6, 10, 11]. Most norovirus genotype studies in Korea have focused only on the capsid, and not polymerase genotypes, and no studies addressed the amino acid composition of the norovirus capsid.

The purpose of this study was to investigate the molecular epidemiology of norovirus at the level of polymerase and capsid genes and to characterize the most frequently detected norovirus strain in Korea in 2013 using phylogenetic, similarity, and capsid epitope analyses.

## 2. Methods

### 2.1. Sample Collection

This study was approved by the Institutional Review Board of Hallym University Dongtan Sacred Heart Hospital (IRB number 2013-030). A total of 2,980 fecal samples from several cities in the Seoul metropolitan area were collected between January and December 2013 and were tested for norovirus antigen at the Department of Laboratory Medicine, Hallym University Dongtan Sacred Heart Hospital. Of these, 349 samples (11.7%) yielded positive results with the RIDASCREEN Norovirus antigen test kit (R-Biopharm, Darmstadt, Germany). Norovirus antigen-positive samples were diluted to a 10% stool suspension in phosphate-buffered saline and stored at −70°C. Some norovirus antigen-positive samples could not be further genotyped because the stool volume was insufficient for additional testing or there was PCR failure. Thus, 230 samples were successfully genotyped between January 2013 and December 2013. For these 230 genotyped norovirus samples, the overall median patient age was 1.7 years (range, 1 week to 74 years), and 193 samples (83.9%) were from patients aged less than 5 years.

### 2.2. Norovirus PCR and Sequencing

Viral RNA was extracted from 140 *μ*L of the fecal supernatant using a QIAamp Viral RNA Mini Kit according to manufacturer instructions (Qiagen, Hilden, Germany). All primer sets used for norovirus genotyping are summarized in Table 1.

For norovirus polymerase (ORF1, RdRp) genotyping, either one-step RT-PCR or nested RT-PCR was performed using specific primer sets targeting ORF1 encoding polymerase (RdRp): JV12/JV13 [12] or 1st PCR NV32/NV36 and 2nd PCR NV33/NV35 [13].

For norovirus capsid (ORF2, VP1) genotyping, nested RT-PCR was performed as described previously [5]. One-step RT-PCR was performed using the specific primers GI-F1M/GI-R1M and GII-F1M/GII-R1M, which target ORF2 encoding capsid protein (VP1). The second nested PCR was performed with the primers GI-F2/GI-R1M and GII-F3/GII-R1M.

For phylogenetic, similarity, and epitope analyses of the most frequently detected norovirus strain, one-step RT-PCRs targeting the ORF1-ORF2 junction and remaining ORF2 capsid region were performed using specific primers: NV33/G2SKR [14] and GII-F1M/GV132 [15].

PCR products were visualized by agarose gel electrophoresis and analyzed by DNA sequencing. The nucleotide sequences were analyzed using ABI Prism BigDye Terminator version 3.1 (Applied Biosystems, Foster City, CA, USA). Genotyping of the sequences obtained was performed with an automated genotyping tool (available at http://www.rivm.nl/mpf/norovirus/typingtool [16]) and GenBank nucleotide BLAST search (http://blast.ncbi.nlm.nih.gov/).

### 2.3. Phylogenetic and Similarity Analyses

We performed phylogenetic and similarity analyses to determine the relationship between our most frequently detected norovirus GII.4 strains and representative GII.4 strains. Seven norovirus sequences from GII.4 strains obtained in this study and representative reference GII.4 strains (Farmington Hills 2002/USA/AY502023, Hunter 2004/AU/DQ078814, Den Haag 2006/NL/EF126965, Apeldoorn 2007/AB445395, New Orleans 2009/USA/GU445325, Sydney 2012/AU/JX459908, and Sydney 2012/KR/KM272334), including the closest strain (Sydney 2012/Taiwan/KJ533134), were selected and aligned with MEGA version 6. Reference strain sequences were retrieved from GenBank. The 4331-6660 nucleotide regions in ORF1-ORF2 (polymerase-capsid) region were compared. Phylogenetic and similarity analyses were performed to evaluate the genetic relationships between these sequences. Sites with ambiguous alignments were removed before phylogenetic analysis. The phylogenetic trees were constructed using the neighbor-joining method and different substitution models depending on the genomic region studied, with 1000 bootstraps. Similarity percentages between sequences and reference sequences were assessed with the SimPlot program version 3.5.1.

### 2.4. Comparison of the Capsid Antigenic Epitopes of the Strains from This Study with Reference Strains

Deduced amino acid sequences of norovirus capsid antigens were analyzed and compared to the reference strains. The capsid hypervariable P2 antigenic epitopes (A to E) of the norovirus strains obtained from this study were compared to the reference strains. Multiple sequence alignment and translation to amino acids were conducted using the MEGA version 6 program.

## 3. Results

### 3.1. Monthly Norovirus Antigen Positivity Rates

The rates of norovirus antigen positivity between January 2013 and December 2013 are shown by month in Table 2. During 2013, the monthly norovirus antigen positivity rates showed a V-shaped distribution between January (23.6%) and December (32.0%), with the lowest rate detected in August (1.8%). Norovirus infections were observed throughout the year. Positivity rates surpassed 20% in January and in November to December 2013.

### 3.2. Norovirus Genotype

Of the 230 genotyped samples, norovirus GI was detected in four (1.7%) cases and GII in 226 (98.3%) cases (Table 3). Capsid genotyping results showed that GII.4 (77.4%) was the most frequent genotype, followed by GII.3 (6.1%), GII.13 (3.9%), GII.2 (2.6%), GII.6 (2.6%), GII.17 (2.6%), and GII.21 (1.7%). GII.4 Sydney 2012, which has been reported in Australia, New Zealand, Japan, Western Europe, Canada, and the USA, was the predominant genotype during this period, comprising 164/177 norovirus GII.4-positive samples (92.7%), and was detected throughout 2013, except June and July, and its frequency increased in November and December (Table 2). Four samples (1.7%) were of the norovirus GII.4 Den Haag 2006b genotype (Table 3). GII.Pe/GII.4 Sydney 2012 was the most frequent polymerase/capsid genotype (65.7%), followed by GII.P17/GII.17 (2.1%), GII.P21/GII.3 (2.1%), GII.P16/GII.2 (1.7%), GII.P16/GII.13 (1.3%), GII.P4 2006b/GII.4 2006b (0.9%), and GII.P21/GII.21 (0.9%). GII.P17/GII.17 was the second most frequently detected polymerase/capsid genotype in this study. BLAST searches revealed that GII.P17/GII.17 strains in this study, isolated from January to April 2013, were closest to norovirus Hu/GII/CN/2013/GII.P17_GII.17/Nanjing010141 isolated in China in June 2013 and norovirus Hu/GII/JP/2013/GII.P17_GII.17/Saitama5309 isolated in Japan in 2013.

The genotype frequencies of GII.4 Sydney and GII.4 were significantly higher in the group of children less than 5 years of age than in the group of people older than 5 years (79.3% (153/193) versus 56.8% (21/37), *P* < 0.05). Other genotype frequencies were not significantly different between these two groups.

### 3.3. Phylogenetic and Similarity Analyses

**Figure 1:**
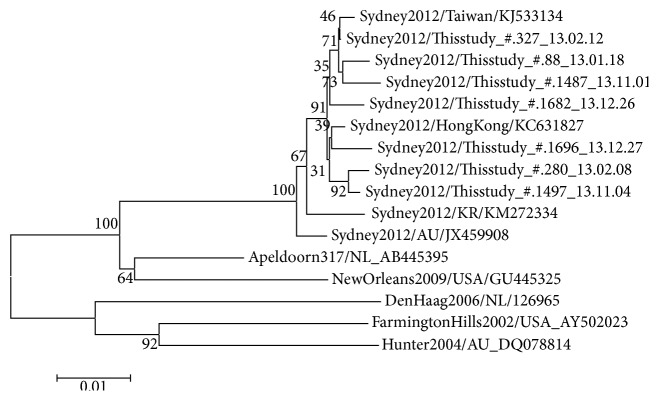
Phylogenetic tree based on partial nucleotide sequences of norovirus GII.4 ORF1 and ORF2. Seven samples of GII.Pe/GII.4 Sydney 2012 genotype in this study were compared to other representative GII.4 reference strains. The sequences of the region comprising nucleotide 4331-6660 were compared. The scale bar represents genetic distance (expressed as nucleotide substitutions per site).

**Figure 2:**
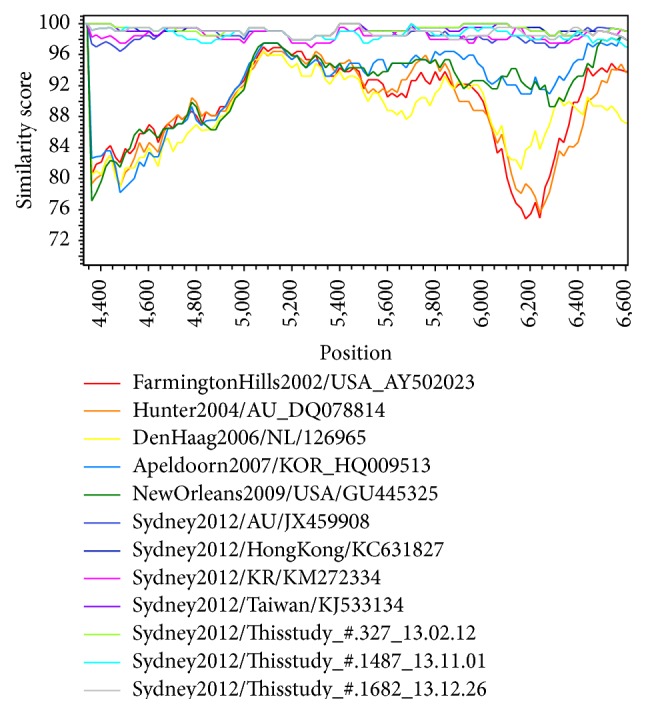
SimPlot analysis for nucleotide similarity between the GII.4 Sydney strains in this study and other previously reported GII.4 Sydney strains and other GII.4 variants causing pandemics. The sequence of one strain in this study served as a query strain. The sequences of the regions comprising nucleotide 4331-6660 were compared.

We randomly selected seven samples of GII.Pe/GII.4 Sydney 2012, the most frequent polymerase/capsid genotype collected in 2013, and compared the 4331-6660 nucleotide sequence regions in ORF1-ORF2 (polymerase-capsid). In all samples, the norovirus genotyping tools yielded GII.Pe/GII.4 variant Sydney 2012 as the closest sequence. BLAST analysis identified norovirus 13-Z-2/2012/GII.4 (GenBank accession number KJ533134) as the closest strain (query coverage = 100%, identity = 99%), obtained in Taiwan in February 2013, compared to one of the strains in this study (strain #1487 isolated in February 2013). The identities between this strain (13-Z-2/2012/GII.4) and the strains in our study were 99.1–99.6%. Phylogenetic trees and SimPlot analysis showed genetic distances among the norovirus reference strains (Figures 1 and 2).

### 3.4. Comparison of Capsid Antigen Epitopes

The deduced amino acid sequences of the A–E epitopes of the capsid P2 domain of representative reference GII.4 strains and the strains in this study are shown in Table 4. The amino acids of A–E epitopes of the norovirus strains analyzed in this study were very similar between the strains, with the exception of residues 373 (beside epitope A) and 393 (in epitope D). The original amino acid at position 393 of norovirus GII.4 Sydney 2012 was glycine (G), detected in some of our strains. Serine (S) was encoded at this position in other strains, which was the same amino acid in the New Orleans 2009 strain.

## 4. Discussion

In this study, we investigated the epidemiology of norovirus infections and the genetic diversity of norovirus strains circulating in metropolitan areas of Korea in 2013. Norovirus infections varied seasonally (monthly positive rate: 1.8~32.0%) and the number of norovirus infections in this study increased in late autumn and winter (January, November, and December), similar to the findings of previous studies in Korea [8] and other countries north of the equator (http://www.cdc.gov/norovirus/). In countries south of the equator, norovirus activity increases around August, also in winter (http://www.cdc.gov/norovirus/). Thus, norovirus infection is most common in cold seasons.

The most frequently detected norovirus polymerase/capsid genotype in this study was classified as GII.Pe/GII.4 Sydney 2012. The GII.4 Sydney 2012 capsid genotype, first reported in Australia in 2012, caused the global outbreak of 2012-2013 [3] and replaced the GII.4 New Orleans 2009 strain. The GII.Pe polymerase genotype was first detected in the norovirus outbreak of 2008 in Victoria, Australia, and then at lower frequencies in 2009 and 2010 before becoming the predominant genotype in 2012 [17]. GII.Pe was linked to the GII.3, GII.4, and GII.12 capsid genotypes [17]. Thus, GII.Pe/GII.4 Sydney 2012 is a recombinant of GII.Pe polymerase and GII.4 capsid gene variant sharing sequences with Apeldoorn 2008 and New Orleans 2009 [3].

BLAST searches revealed that norovirus 13-Z-2/2012/GII.4 collected in Taiwan in February 2013 was closest to a GII.Pe/GII.4 Sydney 2012 isolated from this study, also collected in February 2013. Thus, similar GII.Pe/GII.4 Sydney 2012 strains were active in Korea and Taiwan at the same time. The identities between this strain (13-Z-2/2012/GII.4) and the GII.Pe/GII.4 Sydney 2012 strains identified in our study were 99.1–99.6%. Thus, similar GII.Pe/GII.4 Sydney 2012 strains were the main cause of norovirus infection in 2013.

Recombination in the region between ORF1 polymerase and ORF2 capsid is common in norovirus infection. The polymerase-capsid recombination of GII.P16/GII.2, GII.P21/GII.3, GII.P12/GII.3, GII.P16/GII.13, and GII.Pe/GII.3 found in this study has been reported elsewhere [17, 18]. The GII.P17/GII.17 was the second most frequently detected polymerase/capsid genotype in this study. BLAST searches revealed that GII.P17/GII.17 strains in this study, isolated from January to April 2013, were closest to norovirus Hu/GII/CN/2013/GII.P17_GII.17/Nanjing010141 isolated in China in June 2013 and norovirus Hu/GII/JP/2013/GII.P17_GII.17/Saitama5309 isolated in Japan in 2013. Thus, similar GII.P17/GII.17 strains were active in Korea, China, and Japan in 2013. The sequences of these strains were slightly different from those of a novel GII.P17-GII.17 norovirus strain (GII.17 Kawasaki 2014), which emerged as a major cause of gastroenteritis outbreaks in China and Japan in the winter of 2014/2015 [19]. From these findings, we can assume that a novel escape mutant derived from the 2013 GII.P17-GII.17 strains in this study emerged and caused gastroenteritis outbreaks in China and Japan in the winter of 2014/2015.

The genotype frequency of GII.4 Sydney or GII.4 was significantly higher in the group of children aged less than 5 years than in the group of people older than 5 years [79.3% (153/193) versus 56.8% (21/37), *P* < 0.05]. Other genotypes were not significantly different between the two age groups. This could be because GII.4 Sydney norovirus was predominant during this period, and the immunity of children to norovirus infection is weaker than that of adults; furthermore, the children usually stayed in childcare facilities in Korea.

Antigenic variation in GII.4 is an important contributor to the emergence of novel pandemic noroviruses [20], with most variants appearing in five evolving blockade epitopes (A–E) within the capsid P2 domain [7]. In this study, the A–E epitopes were similar with the exception of the residues at positions 373 (adjacent to epitope A) and 393 (epitope D). Mutations at positions 333, 340, and 393 of the capsid protein likely played a role in the evolution/adaptation or escape from herd immunity to the novel pandemic GII.4 variant [21]. It is also possible that other, not yet identified, mechanisms were also involved. Further studies on the evolutionary pathway of new GII.4 variants will be needed to predict the emergence of new pandemic strains and to guide vaccine development.

## 5. Conclusions

In summary, increased norovirus activity in Korea in 2013 was related to the emergence of a norovirus GII.4 variant, GII.Pe/GII.4 Sydney 2012, similar to the findings in global epidemics. Epitope analysis showed at least one amino acid difference in comparison to previous GII.4 reference strains. These data improve our knowledge in relation to viral evolution, outbreak predictions, and vaccine development. As new GII.4 variants emerge every 2-3 years and cause global epidemics, continuous monitoring of new norovirus GII.4 variants will be needed to predict the emergence of new pandemic strains and to guide vaccine development.

## Figures and Tables

**Table 1:** Primers used in this study for the RT-PCR and sequencing of norovirus.

Targeted gene		Reaction type	Primer name	Primer orientation	Primer sequence (5′ to 3′)	Annealing position	PCR product size
ORF1 (polymerase, RdRp)	1	One-step RT-PCR	JV12	F	ATA CCA CTA TGA TGC AGA TTA	nt 4552	327 bp
			JV13	R	TCA TCA TCA CCA TAG AAA GAG	nt 4878	
	2	1st PCR	NV32	F	ATG AAT ATG AAT GAA GAT GG	nt 4226	501 bp
			NV36	R	ATT GGT CCT TCT GTT TTG TC	nt 4726	
		Nested PCR	NV33	F	TAC CAC TAT GAT GCA GAT TA	nt 4280	357 bp
			NV35	R	GTT GAC ACA ATC TCA TCA TC	nt 4636	

ORF2 (capsid, VP1)	1 (GI)	1st PCR	GI-F1M	F	CTG CCC GAA TTY GTA AAT GAT GAT	nt 5342	330 bp
			GI-R1M	R	CCA ACC CAR CCA TTR TAC ATY TG	nt 5671	
		Nested PCR	GI-F2	F	ATG ATG ATG GCG TCT AAG GAC GC	nt 5357	315 bp
			GI-R1M	R	CCA ACC CAR CCA TTR TAC ATY TG	nt 5671	
	2 (GII)	1st PCR	GII-F1M	F	GGG AGG GCG ATC GCA ATC	nt 5058	344 bp
			GII-R1M	R	CCR CCT GCA TRI CCR TTR TAC AT	nt 5401	
		Nested PCR	GII-F3	F	TTG CCR GCA TRI CCR TTR TAC AT	nt 5088	314 bp
			GII-R1M	R	CCR CCT GCA TRI CCR TTR TAC AT	nt 5401	

ORF1-ORF2 junction		One-step RT-PCR	NV33	F	TAC CAC TAT GAT GCA GAT TA	nt 4280	1110 bp
			G2SKR	R	CCR CCN GCA TRH CCR TTR TAC AT	nt 5389	

ORF2 (capsid, VP1)		One-step RT-PCR	GII-F1M	F	GGG AGG GCG ATC GCA ATC	nt 5049	1681 bp
			GV132	R	CCR GCR AAG AAA GCR CCA GCC AT	nt 6729	

F, forward; R, reverse.

**Table 2:** Norovirus antigen positivity and GII.4 strain frequencies by month.

Date	Number (No.) of specimens tested	Number (%) of norovirus antigen-positive specimens	Number of genotyped specimens	Number (%) of GII.4 strains of genotyped specimens	Number (%) of GII.4 Sydney 2012 strains of genotyped specimens
Jan. 2013	225	53 (23.6%)	33	25 (75.8%)	22 (66.7%)
Feb.	300	38 (12.7%)	28	19 (67.9%)	14 (50.0%)
Mar.	252	27 (10.7%)	17	8 (47.1%)	7 (41.2%)
Apr.	256	16 (6.3%)	10	3 (30.0%)	3 (30.0%)
May	247	11 (4.5%)	2	1 (50.0%)	1 (50.0%)
Jun.	218	6 (2.8%)	1	0 (0%)	0 (0%)
Jul.	237	5 (2.1%)	2	0 (0%)	0 (0%)
Aug.	225	4 (1.8%)	1	1 (100%)	1 (100%)
Sep.	260	11 (4.2%)	8	8 (100%)	7 (87.8%)
Oct.	138	8 (5.8%)	5	3 (60.0%)	3 (60.0%)
Nov.	284	62 (21.8%)	49	47 (95.9%)	47 (95.9%)
Dec.	338	108 (32.0%)	74	62 (83.4%)	59 (79.7%)
Total	2,980	349 (11.7%)	230	177 (77.0%)	164 (71.3%)

**Table 3:** Norovirus polymerase and capsid genotypes.

Polymerase (RdRp) genotype	Capsid (VP1) genotype	Number (%)
Nontyped	GI.3	1 (0.4%)
GI.P4	GI.4	1 (0.4%)
Nontyped	GI.6	2 (0.9%)
GII.P16	GII.2	4 (1.7%)
Nontyped	GII.2	2 (0.9%)
GII.P21	GII.3	5 (2.1%)
GII.P12	GII.3	1 (0.4%)
GII.Pe	GII.3	1 (0.4%)
Nontyped	GII.3	7 (3%)
GII.P4 Den Haag 2006b	GII.4 Den Haag 2006b	2 (0.9%)
Nontyped	GII.4 Den Haag 2006b	2 (0.9%)
GII.Pe	GII.4 Sydney 2012	151 (65.7%)
Nontyped	GII.4 Sydney 2012	14 (6.1%)
GII.Pe	GII.4	5 (2.1%)
Nontyped	GII.4	4 (1.7%)
Nontyped	GII.5	1 (0.4%)
Nontyped	GII.6	6 (2.6%)
GII.P8	GII.8	1 (0.4%)
Nontyped	GII.8	1 (0.4%)
GII.P16	GII.13	3 (1.3%)
Nontyped	GII.13	6 (2.6%)
GII.P17	GII.17	5 (2.1%)
Nontyped	GII.17	1 (0.4%)
GII.P21	GII.21	2 (0.9%)
Nontyped	GII.21	2 (0.9%)
	Total	230 (100%)

**Table 4:** Deduced amino acid sequence of capsid antigen epitopes A to E of the norovirus GII.4 variants.

GII.4 variant	Epitope A							Epitope B		Epitope C		Epitope D			Epitope E		
	294	296	297	298	368	372	373	333	382	340	376	393	394	395	407	412	413
Farmington Hills 2002/USA/AY502023	A	T	H	N	N	N	N	M	K	G	E	N	G	A	S	T	G
Hunter 2004/AU/DQ078814	A	A	Q	N	S	S	N	V	R	R	E	S	T	T	D	D	S
Den Haag 2006b/NL/EF126965	A	S	R	N	S	E	N	V	K	G	E	S	T	T	S	N	V
Apeldoorn 2007/NL/AB445395	T	S	R	N	A	D	N	V	K	T	D	D	T	A	S	N	N
New Orleans 2009/USA/GU445325	P	S	R	N	A	D	N	V	K	T	E	S	T	T	S	N	I

Sydney 2012/AU/JX459908_2012.03	**T**	S	R	N	**E**	D	**R**	V	K	T	E	**G**	T	T	S	N	**T**
Sydney 2012/KR/KM272334_12.08.04	**T**	S	R	N	**E**	D	**H**	V	K	T	E	**G**	T	T	S	N	**T**
Sydney 2012/Hong Kong/KC631827_12.12.10	**T**	S	R	N	**E**	D	**H**	V	K	T	E	**S**	T	T	S	N	**T**
Sydney 2012/Taiwan/KJ533134_13.02	**T**	S	R	N	**E**	D	**R**	V	K	T	E	**S**	T	T	S	N	**T**
Sydney 2012 in this study #88_13.01.18	**T**	S	R	N	**E**	D	**R**	V	K	T	E	**G**	T	T	S	N	**T**
Sydney 2012 in this study #280_13.02.08	**T**	S	R	N	**E**	D	**R**	V	K	T	E	**S**	T	T	S	N	**T**
Sydney 2012 in this study #327_13.02.12	**T**	S	R	N	**E**	D	**R**	V	K	T	E	**S**	T	T	S	N	**T**
Sydney 2012 in this study #1487_13.11.01	**T**	S	R	N	**E**	D	**R**	V	K	T	E	**G**	T	T	S	N	**T**
Sydney 2012 in this study #1497_13.11.04	**T**	S	R	N	**E**	D	**R**	V	K	T	E	**S**	T	T	S	N	**T**
Sydney 2012 in this study #1682_13.12.26	**T**	S	R	N	**E**	D	**R**	V	K	T	E	**G**	T	T	S	N	**T**
Sydney 2012 in this study #1696_13.12.27	**T**	S	R	N	**E**	D	**H**	V	K	T	E	**S**	T	T	S	N	**T**
